# Supplementary material for: The prevalence and common risk indicators of root caries and oral health service utilization pattern among adults, a cross-sectional study
Source: PeerJ. 2023 Nov 22;11:e16458. doi: 10.7717/peerj.16458 (PMC10676080; doi:10.7717/peerj.16458)
Supplement: Supplemental Information 1 [file peerj-11-16458-s001.docx]

Checklist

Upper jaw root status and Mandibular root status

0 ：no caries

1：root caries

2：filled with caries

3：fillecd without caries

6：Residual root

7：implant

8：not exposed

9：do not record

Maxillary periodontal pocket and Mandibular periodontal pocket

0：no pocket more than 3mm

1：4-5mm

2： more than 6mm

9：do not record

X：tooth loss

Maxillary Loss of attachment and Mandibular loss of attachment

0：0-3mm

1： 4-5mm

2：6-8mm

3：9-11mm

4：more than 12mm

9：do not record

X：tooth loss

Questionnaire：

Frequency of eating sweet desserts and sweets& Frequency of sweet beverage& Frequency of milk, yogurt, milk powder, tea, soy milk, coffee

6：≥twice everyday

5：once everyday

4：once every week
3：once every week

2：1-3times every month

1：never/ seldom

smoking

1：yes

2：never

3：quit smoking

How many cigarettes are smoked per day?

1：1

2：1-5

3：6-10

4：11-20

5：21-4-

6：more than 41

alcohol intake

1：every day

2：every week

3：seldom

4：never

5：quit alcohol

Brushing frequency& Toothpick frequency& Floss frequency

6：≥twice everyday

5：once everyday

4：once every week
3：once every week

2：1-3times every month

1：never/ seldom

Do you use toothpaste to brush your teeth?

1：yes

2：no

3：I don‘t know

Do you brushing your teeth with fluoride toothpaste?

1：yes

2：no

3：I don‘t know
