# Supplementary material for: The prevalence and common risk indicators of root caries and oral health service utilization pattern among adults, a cross-sectional study
Source: PeerJ. 2023 Nov 22;11:e16458. doi: 10.7717/peerj.16458 (PMC10676080; doi:10.7717/peerj.16458)
Supplement: Supplemental Information 3 [file peerj-11-16458-s003.pdf]

The 4th National Oral Health Questionnaire 2015 (Adult)

2015 年第四次全国口腔健康调查问卷（成人）

Respondent ID number

被调查者 ID 号：□□□□□□□□□□□□

respondent name

被调查者姓名：__________

date

调查日期：201□年□□月□□日

调查员编号:□ investigator number

要求：请在选择题相应选项前面的“□”内划“√”。

Requirement: Please mark "√" in the "□" in front of the corresponding option of the multiple-choice question.

1. 您的最高学历是?（**只选一个答案**） What's your 'highest degree?

1）□没有上过学 never　2）□小学 primary school　3）□初中 junior high school　4）□高中 High school

5）□中专 Secondary school　6）□大专 college　7）□本科 Bachelor　8）□硕士及以上 Master degree and above

2. 您平时进食以下食品或饮料的频率如何?（**每小题选一个答案**）

How often do you eat the following foods or drinks? (one answer per question)

	6 每天 ≥2 次 everyday	5 每天 1 次 everyday	4 每周 2-6 次 every week	3 每周 1 次 every week	2 每月 1-3 次 every month	1 很少/从不 never/ seldom
1）甜点心（饼干、蛋糕、面包）及糖果（巧克力、含糖口香糖） Sweet snacks (biscuits, cakes, breads) and Candy (chocolate, sugary gum)	□	□	□	□	□	□
2）甜饮料（糖水、可乐等碳酸饮料，橙汁、苹果汁等果汁，柠檬水等非鲜榨果汁） Sweet drinks (sweet drinks such as sugar water, cola, juices such as orange juice and apple juice, non-fresh juices such as lemonade)	□	□	□	□	□	□
3）加糖的牛奶、酸奶、奶粉、茶、豆浆、咖啡 Sugared milk, yogurt, milk powder, tea, Soy milk, coffee	□	□	□	□	□	□

3. 您吸烟吗？（**只选一个答案**） Do you smoke?

1）□吸烟 yes　2）□从不吸 never　3）□已戒烟 Quit smoking（**选 2 或 3 项者不回答第 4、5 题**）(If you choose 2 or 3, you will not answer questions 4 and 5.)

4. 您吸烟多少年了？ ____________年。（**请填一个整数，不知道或拒绝回答的填写“N”**） How many years have you been smoking?——years

5. 最近一个月内，您平均每天吸多少支烟？（**只选一个答案**） How many cigarettes do you smoke on average per day in the last month?

1）□ ≤1 支/天　2）□ 1-5 支/天　3）□ 6-10 支/天

4）□ 11-20 支/天　5）□ 21-40 支/天　6）□ ≥41 支/天

number/per day

6. 您喝白酒吗？（**只选一个答案**）Do you drink wine?

1）□每天喝 every day　2）□每周喝 every week　3）□很少喝 seldom　4）□从不喝 never　5）□已戒酒 quit alcohol

7. 您使用下列方法清洁牙齿吗？（**每小题选一个答案**） Do you use the following methods to clean your teeth? (Select one answer for each question)

	6 每天 ≥2 次 ≥2 times per day	5 每天 1 次 once per day	4 每周 2-6 次 2-6 times a week	3 每周 1 次 once a week	2 每月 1-3 次 1-3 times per month	1 很少/从不 never/seldom
1）刷牙 Brushing teeth	□	□	□	□	□	□
2）牙签 toothpick	□	□	□	□	□	□
3）牙线 Dental floss	□	□	□	□	□	□

闪电PDF编辑器
http://bianji.sdpdf.net

Do you use toothpaste to brush your teeth?

8. 您使用牙膏刷牙吗？（**只选一个答案**）

1）□是 2）□否 3）□不知道（**选 2 或 3 项者不回答第 9 题**）

yes no I don‘t know

9. 您使用含氟牙膏刷牙吗？（**只选一个答案**） Are you brushing your teeth with fluoride toothpaste?

1）□是 2）□否 3）□不知道

yes no I don't know

10. 您看过牙吗？（**只选一个答案**） Have you visited your dentist?

1）□看过 2）□从没看过牙（**选 2 项者不回答第 11 至 15 题**）

yes never

11. 您最近一次看牙距现在多长时间？（**只选一个答案**） How long have you been visiting your dentist last time?

1）□ 6 个月以内 2）□ 6 个月至 12 个月（**选 1 或 2 项者不回答第 16 题**）

3）□ 12 个月以上（**选 3 项者不回答第 12 至 15 题**）

in 6 month 6-12months more than 12 months

12. 您最近一次看牙的主要原因是什么？（**只选一个答案**） reason to visit dentist

1）□咨询检查 2）□预防 3）□治疗 4）□不知道

inspection prevention treatment don't know

13. 在过去的一年内您看牙的总费用是 ________ 元？（**请填一个整数，不知道或拒绝回答的填写"N"**）

total cost to pay for dental care，you can refuse to answer filling N

14. 在上述看牙费用中，您个人需要支付的比例是 ________ %。（**请填一个整数，不知道或拒绝回答的填写"N"**）

The proportion you need to pay personally is%you can refuse to answer filling N

15. 您上一次看牙，费用是否可报销？（**可多选**） is the fee reimbursable?

1）□城镇职工基本保险 2）□城镇居民基本医疗保险

3）□新型农村合作医疗 4）□商业保险

5）□公费医疗 6）□其他途径报销

7）□全部自费（没有报销）

different insurances

16. 您过去 12 个月内**没有看过牙的原因**是？（**可多选**） The reason you haven't visit dentists in the past 12 months is?

1）□牙齿没有问题 2）□牙病不重

3）□没有时间 4）□经济困难，看不起牙

5）□看牙不能报销 6）□附近没有牙医

7）□害怕传染病 8）□害怕看牙疼痛

9）□很难找到信得过的牙医 10）□挂号太难

11）□其它原因

reasons. do not translate because it was not the content of my article

17. 您是否有以下的医疗保障？（**每小题选一个答案**） Do you have the following medical coverage?

	是 1	否 2
1）城镇职工基本医疗保险	□	□
2）城镇居民基本医疗保险	□	□
3）新型农村合作医疗	□	□
4）商业保险	□	□
5）公费医疗	□	□

different insurance

闪电PDF编辑器
http://bianji.sdpdf.net

18. 在过去的 12 个月内，您洗过牙吗？ Have you washed your teeth in the past 12 months?

1）□是　　2）□否（**选 2 项者不回答第 19 题**）

yes　　no

19. 您洗牙费用的报销方式是（**可多选**） The reimbursement method for your dental cleaning costs is

1）□城镇职工基本保险　　2）□城镇居民基本医疗保险

3）□新型农村合作医疗　　4）□商业保险

5）□公费医疗　　6）□其他途径报销　　different type of insurance

7）□全部自费（没有报销）

20. 口腔问题对您以下方面的影响有多大？（**55-64 岁年龄组不回答此题，每小题选一个答案**）

How much does the oral problem affect you in the following areas?

no translation because this part of question weren't involved in my article

	1<br>很经常	2<br>经常	3<br>有时	4<br>很少	5<br>无
1）您经常因为牙齿或假牙的原因限制所吃食物的种类和数量吗？	□	□	□	□	□
2）您在咬或咀嚼食物时有困难吗？	□	□	□	□	□
3）您吞咽食物时经常会感到不舒服或困难吗？	□	□	□	□	□
4）您的牙齿或假牙妨碍您说话吗？	□	□	□	□	□
5）您吃东西时经常感到口腔内不舒服吗？	□	□	□	□	□
6）您经常因为牙齿或假牙的原因而限制自己与他人的交往吗？	□	□	□	□	□
7）您经常对您牙齿、牙龈或假牙的外观感到不满意或不愉快吗？	□	□	□	□	□
8）您经常用药物缓解口腔的疼痛或不适吗？	□	□	□	□	□
9）您经常担心或关注您的牙齿、牙龈或假牙的问题吗？	□	□	□	□	□
10）您经常因为牙齿、牙龈或假牙的问题而在别人面前感到紧张或不自在吗？	□	□	□	□	□
11）您经常因为牙齿或假牙的问题而在别人面前吃东西时感到不舒服吗？	□	□	□	□	□
12）您的牙齿或牙龈对冷、热或甜刺激敏感吗？	□	□	□	□	□

How do you think of your overall health?

21. 您对自己的全身健康状况评价如何？（**只选一个答案**）

very good 1）□很好　good 2）□较好　3）□一般 not bad

4）□较差　5）□很差

bad　　very poor

22. 您对自己的牙齿和口腔状况评价如何？（**只选一个答案**） How do you rate your teeth and oral condition?

very good 1）□很好 good 2）□较好　3）□一般

4）□较差　5）□很差　not bad

poor　　very poor

闪电PDF编辑器
http://bianji.sdpdf.net

23. 您对以下说法的看法如何？**（每小题选一个答案）**

whether learn about the oral health knowledge or not?

no translation because this part weren't invlved in my article

	1 同意	2 不同意	8 无所谓	9 不知道
1) 口腔健康对自己的生活很重要	☐	☐	☐	☐
2) 定期口腔检查是十分必要的	☐	☐	☐	☐
3) 牙齿的好坏是天生的，与自己的保护关系不大	☐	☐	☐	☐
4) 预防牙病首先靠自己	☐	☐	☐	☐

24. 你认为下面的说法是否正确？**（每小题选一个答案）**

	1 正确	2 不正确	8 不知道
1） 刷牙时牙龈出血是正常的	☐	☐	☐
2） 细菌可以引起牙龈发炎	☐	☐	☐
3） 刷牙对预防牙龈出血**没有用**	☐	☐	☐
4） 细菌可以引起龋齿	☐	☐	☐
5） 吃糖可以导致龋齿	☐	☐	☐
6） 氟化物对保护牙齿**没有用**	☐	☐	☐
7） 窝沟封闭可保护牙齿	☐	☐	☐
8） 口腔疾病可能会影响全身健康	☐	☐	☐

Have you ever had the following chronic diseases diagnosed by your doctor?

25. 你是否曾经患过由医生确诊过的下列慢性病？**（可多选）**

1） ☐中风 Stroke　　2） ☐糖尿病 diabetes　　3） ☐高血压 hypertension

4） ☐心脏病 heart disease　　5） ☐慢性阻塞性肺部疾病 COPD　　6） ☐其它，请注明________ other

7） ☐没有 none　　8） ☐不知道 dont know

26. 您家里共同生活的有几口人？________ 人**（请填一个整数，拒绝回答的填写“N”）**；

How many people live in your family? ___

27. 您家在过去的 12 个月内的总收入大约是多少？_____万元/年**（请填一个整数，拒绝回答的填写“N”）**。

What is the total income of your family in the past 12 months?

十分感谢您的合作！

Thank you so much for your cooperation!

闪电PDF编辑器
http://bianji.sdpdf.net
